# NeuroGator: A Low-Power Gating System for Asynchronous BCI Based on LFP Brain State Estimation

**DOI:** 10.3390/brainsci16020141

**Published:** 2026-01-28

**Authors:** Benyuan He, Chunxiu Liu, Zhimei Qi, Ning Xue, Lei Yao

**Affiliations:** 1 State Key Laboratory of Transducer Technology, Aerospace Information Research Institute (AIR), Chinese Academy of Sciences, Beijing 100094, China; hebenyuan22@mails.ucas.ac.cn (B.H.); cxliu@mail.ie.ac.cn (C.L.); zhimei-qi@mail.ie.ac.cn (Z.Q.); 2 School of Electronic, Electrical, and Communication Engineering, University of Chinese Academy of Sciences (UCAS), Beijing 100049, China; 3 Lingang Laboratory, Shanghai 200031, China; xuening@mail.ie.ac.cn

**Keywords:** implantable brain–computer interface, asynchronous control, low power, Local Field Potentials, Gated Recurrent Unit

## Abstract

The continuous handling of the large amount of raw data generated by implantable brain–computer interface (BCI) devices requires a large amount of hardware resources and is becoming a bottleneck for implantable BCI systems, particularly for power-constrained wireless systems. To overcome this bottleneck, we present NeuroGator, an asynchronous gating system using Local Field Potential (LFP) for the implantable BCI system. Unlike a conventional continuous data decoding approach, NeuroGator uses hierarchical state classification to efficiently allocate hardware resources to reduce the data size before handling or transmission. The proposed NeuroGator operates in two stages: Firstly, a low-power hardware silence detector filters out background noise and non-active signals, effectively reducing the data size by approximately 69.4%. Secondly, a Dual-Resolution Gate Recurrent Unit (GRU) model controls the main data processing procedure on the edge side, using a first-level model to scan low-precision LFP data for potential activity and a second-level model to analyze high-precision LFP data for confirmation of an active state. The experiment shows that NeuroGator reduces overall data throughput by 82% while maintaining an F1-Score of 0.95. This architecture allows the Implantable BCI system to stay in an ultra-low-power state for over 85% of its entire operation period. The proposed NeuroGator has been implemented in an Application-Specific Integrated Circuit (ASIC) with a standard 180 nm Complementary Metal Oxide Semiconductor (CMOS) process, occupying a silicon area of $0.006{\mathrm{mm}}^{2}$ and consuming 51 nW power. NeuroGator effectively resolves the resource efficiency dilemma for implantable BCI devices, offering a robust paradigm for next-generation asynchronous implantable BCI systems.

## 1. Introduction

Implantable brain–computer interfaces (BCIs) have demonstrated immense clinical potential for restoring motor function and facilitating neural rehabilitation [1,2]. Recent clinical evidence suggests that fully implantable systems are moving toward long-term human applications [3], yet they remain hindered by a critical “power-thermal” bottleneck. To achieve high-fidelity control, modern implantable BCIs typically record broadband signals at high sampling rates (>20 kHz) to detect and decode action potentials (Spikes) [4,5]. However, this high-throughput processing generates significant heat dissipation. In confined intracranial environments, the power density of implanted electronics is strictly limited, because a local tissue temperature rise exceeding 1 °C can trigger irreversible inflammatory responses and neuronal damage [6]. Specifically, satisfying this 1 °C safety limit typically constrains the maximum power budget for wireless neural implants to approximately 7.7 mW [7]. Consequently, minimizing power consumption is not merely an energy-saving requirement but a fundamental safety prerequisite for thermal management.

Since motor intent is sparse in daily life, maintaining high-power decoders in an “always-on” state is highly inefficient. To resolve this, asynchronous BCI strategies—functioning as “brain switches” to wake the system on demand—have emerged as a pivotal solution [8,9]. The engineering challenge lies in selecting a signal modality that balances precision, power, and stability. Within the last three years, various approaches have been explored. Spike-based switches, such as the 384-channel sorting IC proposed by Chen et al. [10], offer high precision but require μW-level power per channel, posing thermal risks in high-density arrays. Similarly, multi-unit activity (MUA)-based systems [11,12,13] achieve robust state estimation but often necessitate computationally intensive learning phases to identify optimal channels. Furthermore, high-frequency spikes are prone to degradation in chronic environments due to glial scarring and micromotion, rendering wake-up systems dependent on them prone to failure [14,15].

Alternatively, low-frequency signals like Spiking Band Power (SBP) and Local Field Potentials (LFPs) offer superior longevity [1,16,17]. Recent work by Atzeni et al. [18] demonstrated low-power SBP recording, yet LFP remains the most robust modality due to its “signal persistence” [8,17]. As demonstrated by Valencia et al. [8], LFP-based switches can achieve performance comparable to spike-based systems with nW-level power [19,20], as they bypass the power-hungry sampling requirements of spikes. LFPs reflect the synchronized activity of neuronal populations, allowing them to remain decodable even after the loss of individual spike signals due to interface encapsulation [21,22]. However, such existing LFP or SBP schemes still face notable limitations in fully implantable applications. First, on-chip processing of full-precision signals for complex frequency band extraction (e.g., parallel bandpass filtering) imposes significant logic complexity and hardware area overhead, while continuous transmission of these features creates persistent pressure on wireless bandwidth and power [23,24]. Second, conventional “Active/Inactive” binary classification logic struggles to capture the intricate transition states between rest and execution; simple switches relying solely on amplitude or frequency thresholds are susceptible to physiological noise, introducing decision errors during critical transition periods [19,24].

Against this backdrop, we propose NeuroGator, a hierarchical low-power gating system for asynchronous BCIs built upon the stability of LFPs. To achieve this, we aim to resolve the trade-off between energy efficiency and longevity in fully implantable devices. Our design allocates data and computational resources on demand. Rather than relying on complex feature engineering, we propose a cascaded processing flow—a “Shallow State Decision Tree” that extends from the hardware substrate to the algorithmic layer. The architecture thus begins with an ultra-low-power on-chip silence detector acting as a gatekeeper, intercepting the vast majority of invalid background noise. Signals passing this screening are processed by a Dual-Resolution Gate Recurrent Unit Cascade (DualGRU). We demonstrate that a lightweight primary model driven by low-precision data (6-bit, 1 kHz) is sufficient to capture ambiguous “transition states”. Only when a final confirmation of the active state is required does the system trigger the high-precision model (12-bit, 2 kHz). Experimental validation demonstrates that NeuroGator reduces data throughput by 82% compared to full-precision systems, while maintaining an F1-Score of 0.95. This architecture allows the BCI to remain in an ultra-low-power state for over 85% of its operating time. In a 180 nm Complementary Metal Oxide Semiconductor (CMOS) process, the core circuit area is just $0.006{\mathrm{mm}}^{2}$. Power consumption is only 51 nW. We believe that NeuroGator provides a robust paradigm for next-generation asynchronous interfaces.

The remainder of this paper is organized as follows: Section 2 details the rationale for using LFPs and the overall NeuroGator architecture. Section 3 presents the experimental setup, results, and discussion, including data preprocessing, feature extraction analysis, and hardware validation. Finally, Section 4 concludes the study and outlines future work.

## 2. Methods and Materials

### 2.1. System Overview

**Figure 1:**
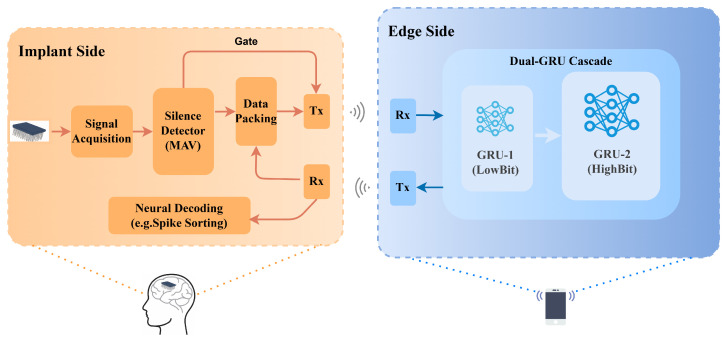
System architecture of the NeuroGator asynchronous gating system.

The NeuroGator system is partitioned into an implantable front-end and an edge decoding unit. Conceptually, its workflow maps to the logic of a “Shallow State Decision Tree.” Building on this, the design philosophy centers on a hierarchical wake-up mechanism to maximize energy efficiency; however, to ensure that this power-saving approach does not compromise the brain-switch’s reliability, we devised a DualGRU model specifically optimized for single-channel processing. As a result, this ensures effective state classification even under strict power constraints. The complete architectural flow is illustrated in Figure 1.

### 2.2. Feasibility and Advantages of Using Local Field Potentials

**Figure 2:**
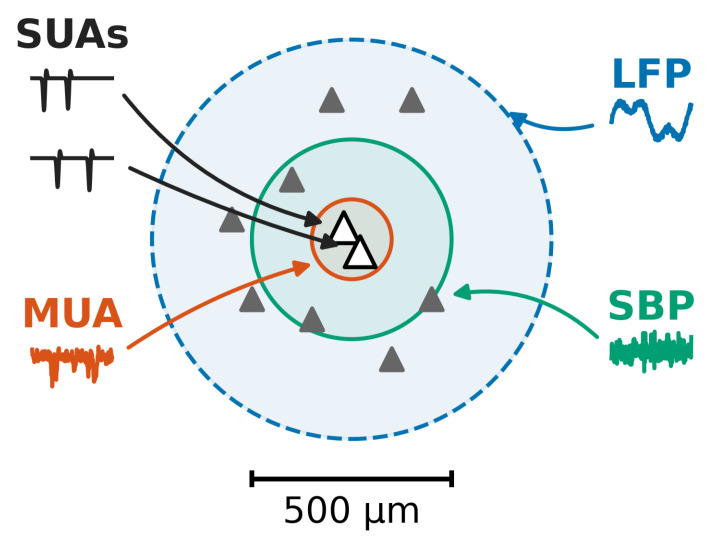
The different signal modalities of intracortical recording electrodes.

Intracortical microelectrode arrays (MEAs) offer a unique window into neural activity across varying spatial scales. Figure 2 illustrates the relative spatial resolution of the alternative signal modalities available for intracortical recording, with the center representing the location of the electrode tip [15]. When an electrode tip sits within a tight 50 μm to 100 μm radius of a neuron, we can successfully isolate single-unit (SUA) or MUA. However, these high-frequency components (>500 Hz) are notoriously sensitive to the immediate recording environment. Local Field Potentials (LFPs) present a contrasting profile. Representing the superposition of transmembrane currents over a range of hundreds of micrometers, LFPs undergo low-pass filtering by the extracellular medium itself. This physically integrates synaptic inputs and sub-threshold oscillations from a local population, offering a much more robust spatial average than the fragile isolation of single spikes. For any system intended for long-term clinical service, signal stability—rather than just instantaneous decoding precision—becomes the defining metric. That emphasis on stability is crucial, as we know that maintaining stable SUA in chronic recordings is an uphill battle; between micromotion and the body’s inevitable tissue response, tracking the exact same neuron over days or weeks is rarely reliable [5,14]. Even more concerning is the irreversible decline in detectable single-neuron yields as implantation time drags on [25,26]. While some researchers turn to Threshold Crossing (TC)-based MUA as a more stable alternative to isolated units, this approach remains an imperfect solution. TC still fundamentally relies on high-frequency components and remains vulnerable to the same signal-to-noise ratio attenuation. In contrast with the ephemeral nature of spikes, LFPs demonstrate remarkable robustness in chronic settings. For example, Flint et al. have clearly shown that LFPs offer superior day-to-day stability compared to threshold crossings [27]. Most notably, the feature known as “signal persistence” is clinically significant: even when glial scarring completely chokes off spike signals—resulting in a total loss of Threshold Crossings—the LFP signal often survives, retaining effective neural decoding capabilities [21,28]. This unique capability to function even after electrode interface degradation positions LFPs as a highly viable candidate for constructing a truly “permanent” wake-up system. Furthermore, from an engineering perspective, shifting to LFPs significantly simplifies the design constraints. Specifically, since LFPs’ sampling and processing rates are a fraction of what is required for spike detection, we can bypass the power-hungry computational overhead of complex spike classification algorithms [10,11]. This, in turn, naturally extends the lifespan of the implanted device [29,30]. Additionally, by relying on LFPs, we might reduce our dependence on deep-penetrating electrodes, potentially mitigating tissue scarring in the first place [29,30].

In summary, spikes provide high-resolution data on individual neuronal firing but are highly sensitive to signal attenuation from glial scarring and electrode micromotion. In contrast, low-frequency LFPs reflect a broader spatial scale and show greater resilience to biological responses after chronic implantation. Information requirements for asynchronous gating differ from those for high-degree-of-freedom kinematic reconstruction. While kinematic reconstruction requires precise spike timing or high-frequency resolution for decoding complex movements, temporal-state detection emphasizes macroscopic population dynamics. Consequently, this task imposes lower demands on fine-grained signal resolution, making LFPs a more suitable and reliable modality for detection in this application. Additionally, using LFPs reduces reliance on deep-penetrating electrodes and greatly lowers the requirements for sampling rates and data transmission bandwidth. Because of their broad spatial integration, robustness, and energy efficiency, LFPs are a pragmatic choice for achieving long-term, stable, low-power, asynchronous brain–computer interface systems.

### 2.3. Shallow State Tree

To balance energy efficiency with decoding accuracy, we discretized continuous neural temporal states into four categories using hierarchical logic. This forms a “Shallow State Tree” that directly maps the NeuroGator decision process. Action states reflected by LFPs are broadly divided into Resting and Active. Resting states are further refined into Distinguishable Resting ($S_{0}$) and Indistinguishable Resting ($S_{1}$). $S_{0}$ marks periods when the subject is fully relaxed, and LFP amplitudes are close to background thermal noise. This state occupies most of the recording. $S_{1}$ covers times when, with no motor intent, high-amplitude interference arises from EMG artifacts, blinking, or other neural fluctuations. These signals physically exceed hardware thresholds. However, they lack the temporal structure of motor preparation. While they may “false wake” the primary system, GRU-1 ultimately rejects them. Conversely, Active states include the Transition State ($S_{2}$) and Fully Active State ($S_{3}$). $S_{2}$ is the dynamic process in a fixed window before the movement trigger point (Switch Release). It represents the climb from resting baseline to active burst. The next state, $S_{3}$, is the stable phase of movement execution and contains complex information. Here, the system enters full-precision mode. GRU-2 uses high-frequency, high-bit-width signals to achieve clear brain-state discrimination.

### 2.4. Level 1: On-Chip Silence Detector

In practical BCI scenarios, neural signals exhibit high temporal sparsity. Valid command windows typically occupy less than 30% of the total operation time, leaving the system in a non-task—or resting—state for over 70% of the day [31]. During these intervals, LFP signals are effectively indistinguishable from low-amplitude background thermal noise. To achieve nanowatt-level standby power, we designed a minimalist detection logic based on the Mean Absolute Value (MAV) of the LFP signal as the system’s first gate. Unlike power calculations requiring energy-intensive multipliers, calculating absolute values requires only simple addition and logic operations, making it ideal for ultra-low-power on-chip implementation independent of external processors. We define the signal strength *E* within a short window $T_{w}$, as shown in Equation (1):

(1) $$E\left[n\right]=\sum_{k=n-T_{w}}^{n}\left|x_{\mathrm{vc}}\left[k\right]\right|$$

**Figure 3:**
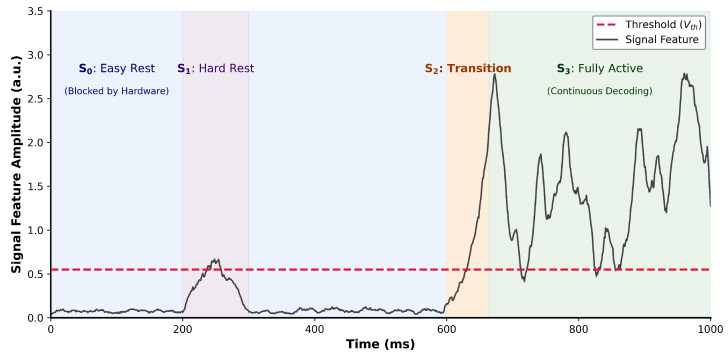
Signal amplitude distributions across four brain states ($S_{0},S_{1},S_{2},S_{3}$) and the determination of the noise threshold ($V_{th}$).

To ensure high sensitivity for system wake-up, the hardware noise threshold, $V_{th}$, was determined by analyzing the probability density function of the signal strength during the Active state $S_{3}$. Instead of defining $V_{th}$ based on the noise floor of the Resting distribution, $V_{th}$ was calculated as the 3% quantile of the Active signal distribution. As shown in Figure 3, this approach ensures that 97% of valid neural activity exceeds the threshold to trigger the system, emphasizing a high recall rate for motor intent over noise rejection.

Operating on this threshold, the system enforces a strict gating protocol. When the windowed $MAV<V_{\mathrm{th}}$, the sample is classified as Distinct Resting ($S_{0}$); since this interval is dominated by background noise, the system determines that no processing is required. Consequently, the back-end wireless transmitter (Tx) and neural codec are completely shut down, leaving only the silence detector active to minimize power draw. Conversely, when $MAV\geq V_{\mathrm{th}}$, the sample is flagged as $S_{1}$ or higher—indicating either potential neural activity or ambiguous noise—which immediately wakes the transmitter to offload the data to the next stage for processing.

### 2.5. Level 2: DualGRU Model

Once signals pass the silence detector, they are transmitted to the edge for precise brain state identification. Selecting the appropriate algorithm is critical for efficient LFP decoding. Although deep learning applications in BCIs have surged [32], and some studies suggest that LFP decoding relies on long-term temporal dynamics [33], practical implementation requires evaluating mainstream RNN variants [34] for LFP time-series processing. These include Long Short-Term Memory (LSTM) [35], Quasi-Recurrent Neural Networks (QRNNs) [36], and Gated Recurrent Units (GRUs) [8]. Standard RNNs, while simple, suffer from vanishing or exploding gradients in long sequences. LSTM addresses long-range dependencies with three gates, but increases parameter count due to its complexity. QRNNs replace direct state propagation with convolutions, allowing speed but sometimes missing complex temporal features. In contrast, GRUs reduce parameter count and simplify computation while preserving decoding accuracy. Based on this comparison, we selected a GRU as the core classifier.

Building on this, we propose a Dual-Resolution Architecture. In the first stage, GRU1 uses 6 bit, 1 kHz LFP signals as input, cutting wireless transmission load by 50% compared to 12 bit, 2 kHz signals. GRU1 classifies Rest vs. Non-Rest, with a focus on high recall for Active states. We prefer false alarms (classifying $S_{1}$ as $S_{2}$) to avoid missing Active states and maintain responsiveness. If GRU1 detects a “Potential Active” state ($S_{2}$ or $S_{3}$), the system switches to high-precision mode. The second stage, GRU2, processes 12 bit, 2 kHz LFP signals to retain fine temporal details and performs high-precision binary classification, filtering GRU1’s false positives to confirm true Active states. The gate is triggered only when GRU2 confirms an Active status, waking the high-power back-end decoder.

## 3. Results and Discussion

### 3.1. Dataset and Experimental Setup

We validated our algorithms using the publicly available rhesus macaque motor cortex dataset [37]. This dataset records neural signals during a spontaneous reach-to-grasp task. It is widely used for benchmarking LFP algorithms [8]. The primary objective of our experimental validation is to verify whether NeuroGator can accurately identify the LFP Active state and maintain ultra-low power consumption by filtering out invalid background noise. To support this, we designed a four-class label generation logic. This maps the experimental task phases to discrete brain states. The actual reach-to-grasp movement, from the movement trigger point until the end of the trial, is labeled as the Fully Active State ($S_{3}$). The period when the monkey is ’waiting’ and ’preparing’ to execute the task is marked as the Transition State ($S_{2}$). Specifically, this is defined as the four consecutive time windows tracing back from the movement preparation point. This division is sufficient to cover the dynamic transition signal from rest to active burst. All remaining non-task intervals are classified as Resting states. These are further divided into Easy Rest ($S_{0}$) and Hard Rest ($S_{1}$), according to the silence detector threshold logic.

The original signals were sampled at 30 kHz to preserve spike details. To reduce edge computing and transmission bandwidth requirements, we downsampled signals to $f_{s}=2\mathrm{kHz}$. This preserves the Delta, Theta, Alpha, Beta, and High-Gamma bands—rich in motor intent information—while drastically cutting data throughput. Furthermore, to enhance robustness in interference-prone environments, we selected N=4 spatially adjacent physical channels on-chip to compute their arithmetic mean, synthesizing a high-quality “Virtual Channel” $x_{\mathrm{vc}}\left(t\right)$:

(2) $$x_{\mathrm{vc}}\left(t\right)=\frac{1}{N}\sum_{i=1}^{N}x_{i}\left(t\right)$$

The reason for using this simple averaging rather than complex beamforming lies in the physical characteristics of microelectrode arrays. Since the electrode spacing is typically small, low-frequency LFP signals (<500 Hz) recorded by adjacent channels have high spatial correlation, and the phase difference is negligible, eliminating the need for high-power phase alignment algorithms. Therefore, simple arithmetic averaging is sufficient to enhance effective neural signal components while effectively canceling out uncorrelated thermal noise and random interference. To train the hierarchical wake-up model, we designed a sample construction process based on a sliding window. The model input is a historical field of view with a length of 512 ms, and the sliding step is set to 16 ms, meaning that the system outputs a decision result every 16 ms. Using the “Switch Release” event in the experimental records as the physical reference point $t_{\mathrm{release}}$ for movement occurrence, we trace back from $t_{\mathrm{release}}$ by $T_{\mathrm{pre}}$ to define the start time of movement preparation.

To comprehensively evaluate the system’s performance on imbalanced datasets, we use the F1-Score as the core metric, which comprehensively considers the model’s precision and recall [8]. All reported performance metrics are presented as mean ± standard deviation (SD) calculated across the five folds. The SD is calculated using the following formula:

(3) $$SD=\sqrt{\frac{\sum_{i=1}^{n}{\left(x_{i}-\bar{x}\right)}^{2}}{n-1}}$$

where n=5, $x_{i}$ represents the performance metric of the *i*-th fold, and $\bar{x}$ denotes the mean value across all folds. Specifically, in the GRU-1 low-power stage, we focus on the recall of the Transition state ($S_{2}$) and Active state, as this metric directly determines the timeliness of the BCI system response and user experience; any missed detection of the Transition state will lead to system wake-up delay. The experiment uses five-fold cross-validation to ensure the reliability of the results. Model training is performed via the Adam optimizer, with the initial learning rate set to $3\times 10^{-4}$, training epochs set to 300, and coupled with an Early Stopping mechanism to prevent overfitting.

### 3.2. Comparison and Selection of On-Chip Feature Extraction Methods

**Figure 4:**
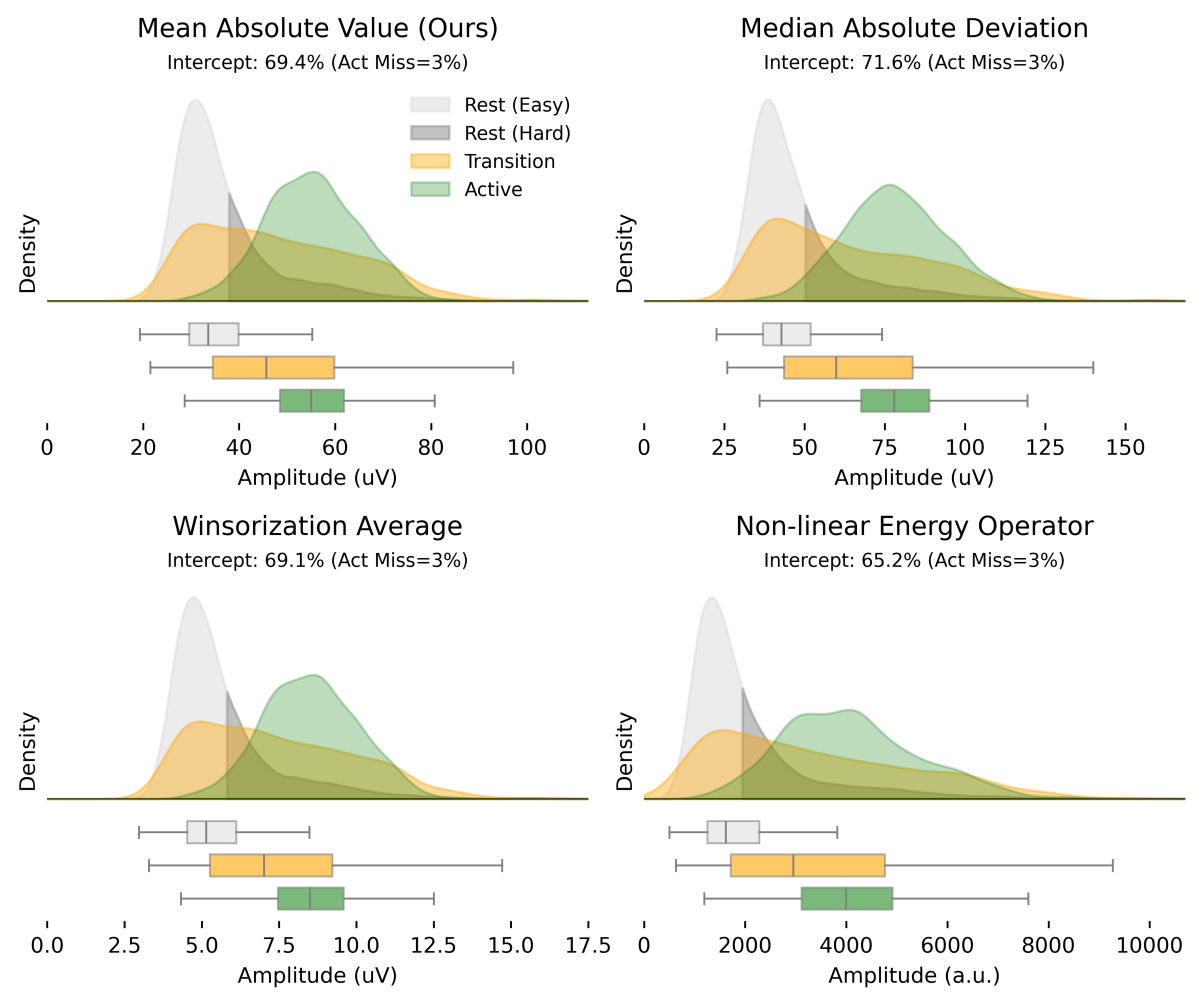
Feature distribution and noise interception performance of different on-chip extraction methods with a fixed 3% active miss rate.

To evaluate the efficacy of the proposed MAV method for LFP state gating, we compared it against three robust feature extraction techniques adopted in neural signal processing: Median Absolute Deviation (MAD) [38], Winsorization Average (WA) [39], and Non-linear Energy Operator (NEO) [40]. As shown in Figure 4, we uniformly constrained the Active Miss Rate to 3% to prioritize system responsiveness. Under this premise, the background noise interception capabilities exhibited distinct trade-offs. The MAD method achieved the highest intercept rate of 71.6%, followed by our MAV method at 69.4%  and WA at 69.1%. The NEO method showed a lower interception rate of 65.2%. While MAD provides the most robust statistical separation, its on-chip implementation necessitates sorting operations on large data arrays, which is computationally prohibitive for ultra-low-power implantable devices. Similarly, the WA method requires additional hardware resources, including comparators for clipping logic and multipliers for scaling coefficients. In contrast, the MAV method achieves comparable interception performance with only simple absolute-value and addition logic. This minimalist architecture enables the hardware layer to intercept 69.4% of invalid data, making it the most hardware-efficient choice for the NeuroGator gating system.

### 3.3. Impact of Channel Count on Model Performance

**Figure 5:**
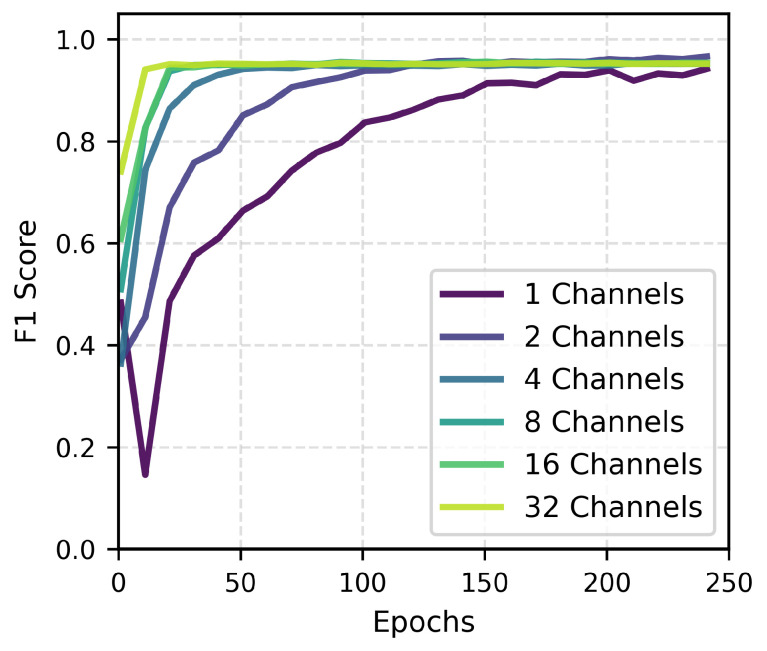
Impact of input channel count on model performance: F1-Score learning curves.

We evaluated the impact of spatial information on LFP active state recognition by comparing GRU model training and performance across input channels of 1, 2, 4, 8, 16, and 32. This allowed us to test whether full-channel input is necessary. The experimental results are shown in Figure 5. The data shows that the accuracy improvement brought by increasing the number of channels exhibits significant diminishing returns. Although the convergence speed of the one-channel input lagged behind that of high-channel configurations—possibly due to the difference in information throughput caused by the reduction in input data dimensions within a single epoch—the final F1-Score differed from the 32-channel full input result by only 0.37%. This result strongly implies that the effective feature information retained in single-channel data is highly consistent with full-channel data; LFP signals have high spatial redundancy, and a few key channels are sufficient to capture macroscopic motor intentions. This conclusion aligns with the findings of Bullard et al. regarding the high correlation between LFP band power and population firing rates [30], while avoiding complex channel selection algorithms [11].

### 3.4. Performance–Efficiency Trade-Off Analysis

**Figure 6:**
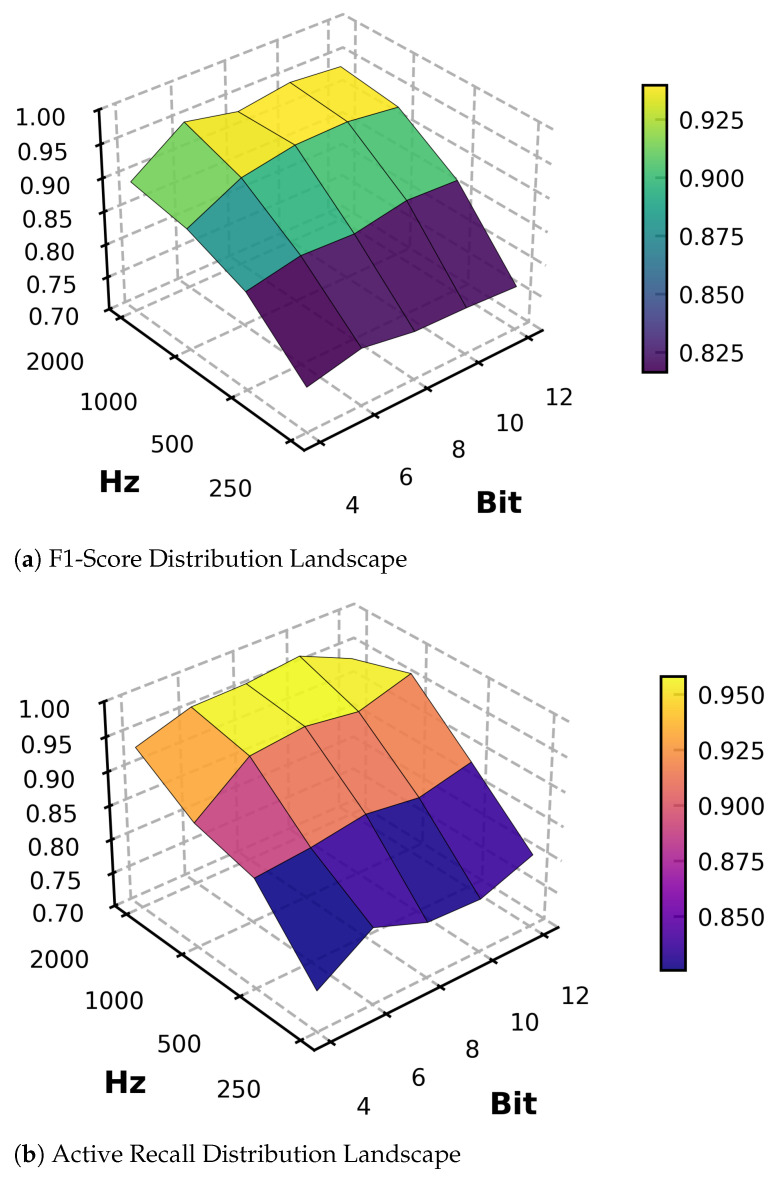
F1-Score heatmap of the GRU model under varying sampling rates and bit-depth configurations.

We investigated the trade-off between LFP active state recognition performance and power consumption for GRU1 in the DualGRU design. A grid search compared model performance across combinations of sampling rates and quantization bit widths. The experimental results are shown in Figure 6. The results reveal that model performance exhibits a clear parameter sensitivity distribution, with the sampling rate being the primary factor affecting accuracy. When the sampling rate is maintained at 1000 Hz and above, the F1-Score generally remains above 0.92, whereas when the sampling rate drops to 500 Hz, the highest F1-Score drops significantly to 0.875, indicating that 1000 Hz is the critical frequency for ensuring signal feature integrity. We analyzed how sampling rate and bit-depth affect the model’s performance. As shown in Figure 6, the model remains robust when the resolution is reduced from 12 bit to 6 bit. However, performance drops significantly when reduced to 4 bit. Based on this, we selected 1 kHz and 6 bit as the optimal configuration. Although this configuration has an accuracy loss of about 1.9% compared to the highest configuration, it achieves a data transmission reduction of up to 75%, and an F1-Score of 0.9225 is sufficient to meet the performance requirements of the GRU1 stage.

**Figure 7:**
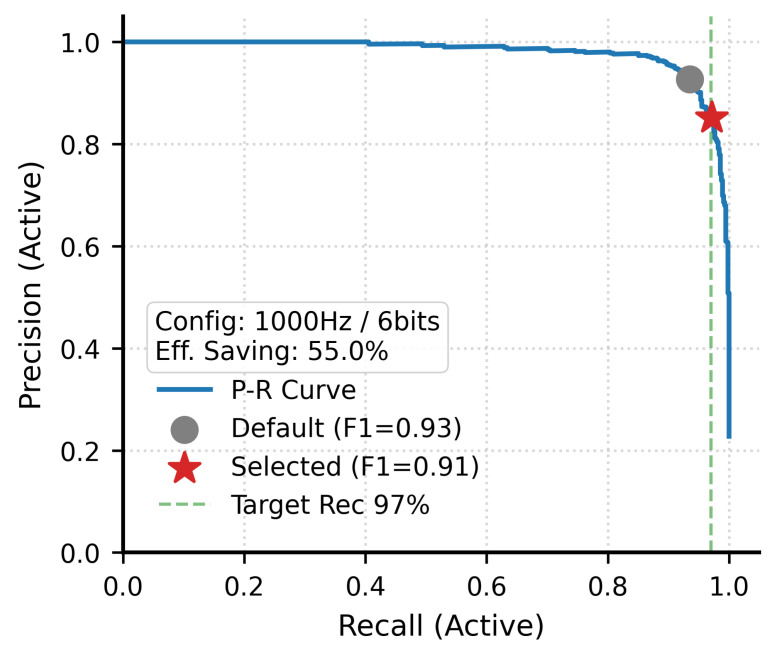
Precision–Recall curve of the GRU1 model using the optimized configuration (1000 Hz/6-bit).

Furthermore, we performed a Precision–Recall trade-off analysis for LFP active state recognition to validate the model’s performance and energy-efficiency advantages under the optimized configuration of 1 kHz and 6 bit (Figure 7). The P-R curve demonstrates that the model can maintain excellent precision in the high Active Recall region. Considering the zero-tolerance characteristic of the wake-up system for missed reports, we adjusted the operating point to a 97% Active target recall rate. Under this threshold, the model can still maintain an F1-Score of 0.91, only slightly lower than the default high-precision configuration. This result strongly supports the feasibility of using low-precision signals as wake-up inputs, meaning that 6 bit coarse-grained LFP waveforms are sufficient to carry macroscopic motor intentions.

The hardware silence detector first intercepts 69.4% of the total recording time, which represents the $S_{0}$ state. Of the remaining 30.6%  of the data, the system spends 55.0% of its duration in the transition or interference states, namely $S_{1}$ and $S_{2}$, where the low-resolution 1 kHz/6-bit configuration is utilized. This leads to an ultra-low-power operating state occupancy defined by the sum of 69.4% and the product of 55% and 30.6%, totaling 86.2%, which is conservatively reported as over 85% of the total operation time. Consequently, the overall reduction in data transmission volume is estimated at 82%, calculated as the summation of the 69.4% hardware interception and the additional algorithmic optimization of 75%×55%×30.6%=12.62%. These efficiencies are fundamental for maintaining thermal safety and extending the battery life of fully implantable BCI devices.

### 3.5. Model Comparison Analysis

**Figure 8:**
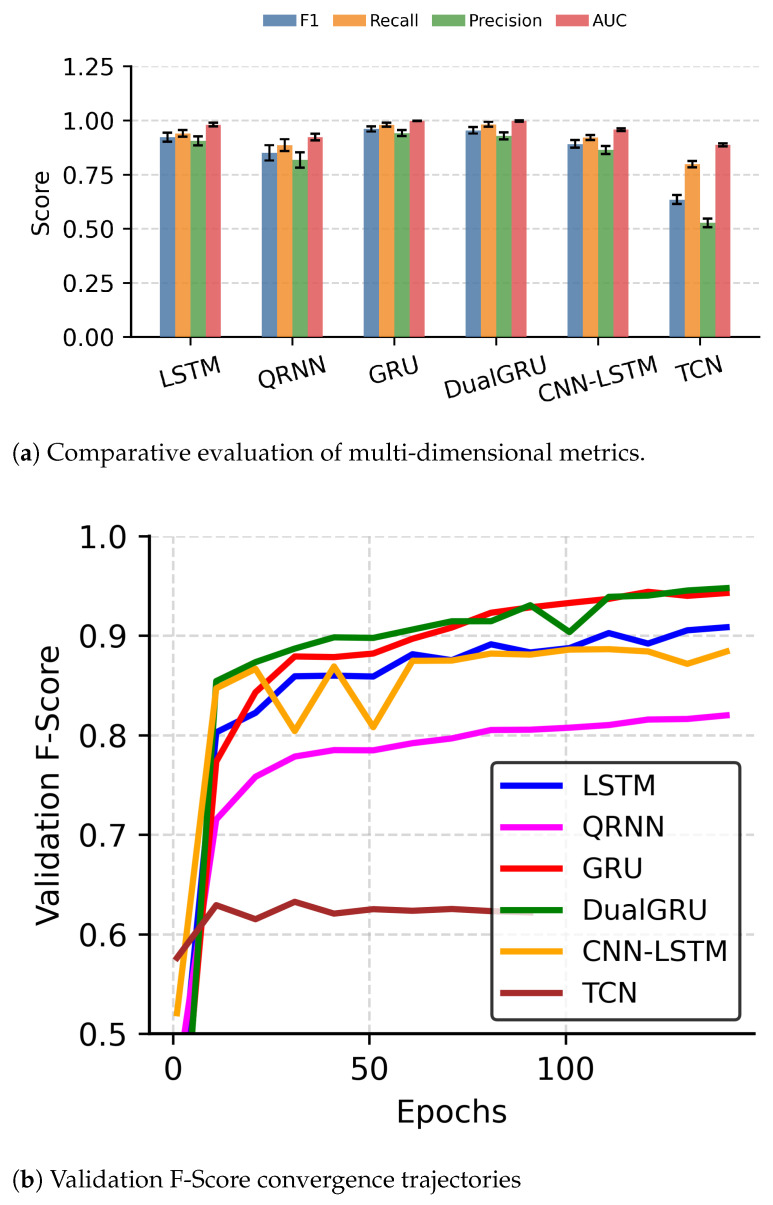
Comprehensive evaluation of model performance and training convergence analysis.

We demonstrate the advantages of the DualGRU architecture for LFP active state recognition and justify using a GRU as the core temporal processing unit by benchmarking our dual-resolution cascaded model against LSTM, QRNN, Convolutional Neural Network (CNN)-LSTM [41], Temporal Convolutional Networks (TCNs) [42], and a single-stage GRU under the same experimental constraints. The detailed comparative experimental results are shown in Figure 8. We compared the parameter count, F1-Score, Recall, Precision, and Area Under the Curve (AUC) metrics of each model. Experimental data show that the single-stage GRU model achieved the highest F1-Score of 0.9603 and AUC of 0.9979. Other architectures exhibited comparatively lower performance: the LSTM model reached an F1-Score of 0.9222, while the CNN-LSTM and QRNN models followed with scores of 0.8914 and 0.8506, respectively. The TCN model underperformed significantly, yielding the lowest F1-Score of 0.6347. This disparity is likely due to the TCN’s limited capacity to capture the specific long-term temporal dependencies inherent in LFP signals when compared to recurrent architectures. This significant performance gap proves that the GRU has extremely high data compression efficiency and feature extraction capability when processing LFP sequences, validating our choice of it as the basic building block.

Although the DualGRU architecture uses low-precision data input in the primary detection stage to significantly reduce front-end wireless transmission power, sacrificing some data precision, thanks to the robust design of the dual-resolution cascaded architecture, its final decoding performance did not decline significantly compared to the full-precision single-stage GRU. Experimental data show that both the single-stage GRU (0.9603±0.012) and the DualGRU (0.9545±0.015) exhibit high statistical stability with minimal fluctuations across cross-validation folds. Compared to the single-stage model, the DualGRU incurs a slight F1-Score loss of less than 0.6%, but it remains superior to other models.

As illustrated in Figure 8b, the architecture demonstrates sufficiently robust generalization capabilities. The integration of the early stopping mechanism, combined with a sufficient number of training epochs, prevents the training process from stagnating near the transient performance dips caused by random noise data, thereby ensuring stable and accurate convergence during model training. Combined with Figure 8b, this architecture successfully demonstrates the feasibility of sacrificing a small amount of non-critical accuracy for significant system-level energy efficiency improvements, representing an optimal solution for low-power scenarios.

### 3.6. System Verification and Comparison

**Figure 9:**
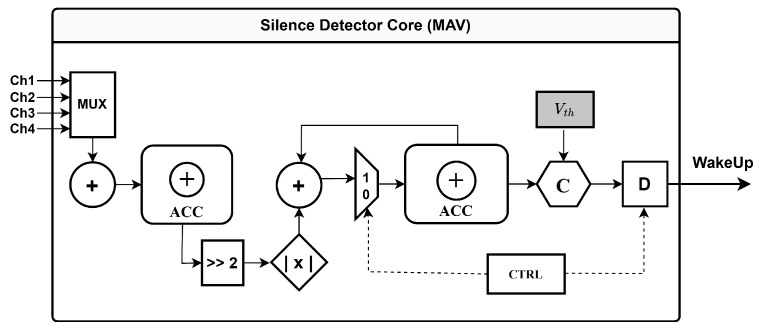
Hardware schematic of the MAV-based silence detector core.

**Figure 10:**
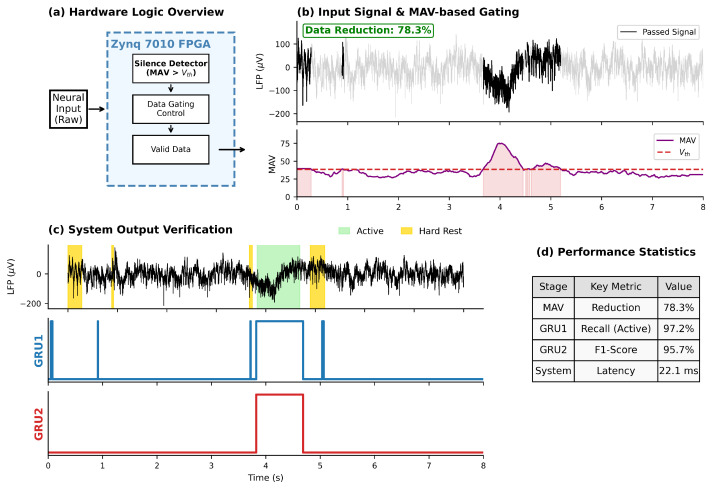
FPGA hardware-in-the-loop validation results and waveform analysis. (**a**) Hardware logic overview on Zynq 7010. (**b**) Input signal and MAV-based gating effect showing 78.3% data reduction. (**c**) System output verification of hierarchical GRU triggering. (**d**) Key performance statistics.

To evaluate the practical efficacy of the NeuroGator system, we implemented the hardware architecture on a Xilinx Zynq 7010 Field-Programmable Gate Array (FPGA) and conducted comprehensive system-level testing. The front-end silence detection core utilizes a hardware logic implementation based on Mean Absolute Value (MAV), comprising a four-channel multiplexer, an accumulator, and a comparator, as detailed in Figure 9. Real-time waveforms captured with a logic analyzer after deployment of the complete processing link are shown in Figure 10. The system demonstrated robust adaptive performance, effectively tracking signal fluctuations and suppressing data transmission during resting intervals. By employing a hierarchical wake-up mechanism—transitioning from coarse-grained detection to fine-grained decoding—the system achieved real-time gating of neural signals with a classification accuracy of 95.7% and a total response latency of less than 22 ms.

**Figure 11:**
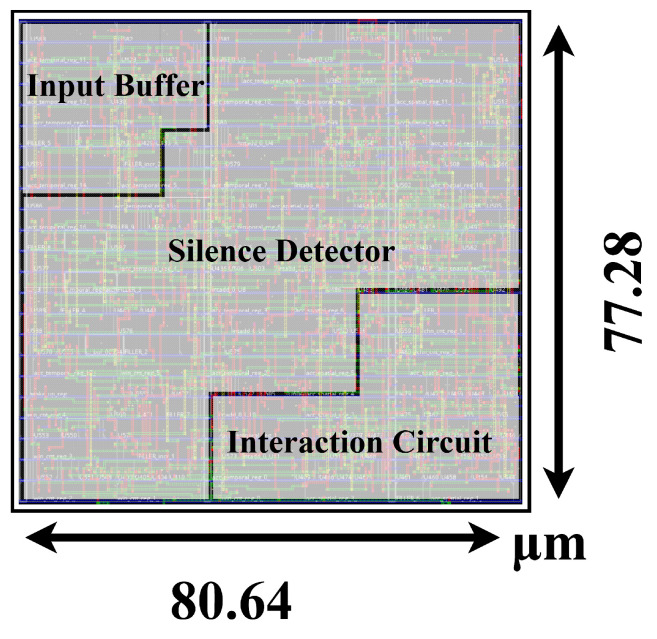
Layout of the proposed NeuroGator feature extraction ASIC.

To assess the feasibility of integrating this architecture into fully implantable systems, we designed an ASIC in a standard 180 nm CMOS process and evaluated its area and power characteristics. Following this, the physical implementation, including placement and routing, was performed using Cadence Innovus. For accurate power estimation, we adhered to standard methodologies from prior studies [8,11], conducting post-simulations on the post-routed netlist. By using a test subset of the dataset as the input stimulus, we captured realistic circuit-switching activity and dynamic power transitions. The design layout and the hardware schematic of the silence detector core are illustrated in Figure 11 and Figure 9, respectively.

The exceptional energy efficiency of NeuroGator is fundamentally rooted in the physical characteristics of the LFP modality. The inherent low temporal resolution of LFP signals relative to action potentials allows for lower sampling rates and narrower bandwidths. This characteristic directly impacts system efficiency: the power consumption of a Low-Noise Amplifier (LNA) is directly proportional to its target bandwidth [29], thus minimizing LNA requirements for LFPs. Similarly, power consumption in a conventional successive approximation register Analog-to-Digital Converter (ADC) scales linearly with the sampling rate [43], making the LFP modality inherently more energy-efficient. By integrating on-chip silence detection and hierarchical quantization, NeuroGator reduces the per-channel data rate from 24 kbps to below 4.8 kbps, significantly decreasing the energy budget for wireless data transmission.

Compared to prior research utilizing MUA features [11,44], which generally necessitate a minimum of eight channels and high sampling rates (∼10 kS/s), resulting in a power consumption of 7.4 μW per channel [45], NeuroGator achieves significant savings. While SBP-based methods [18,46] employ multi-frequency analysis, they still incur higher acquisition costs compared to our cascaded LFP architecture. By operating at 80 kHz, NeuroGator ensures a sufficient computational margin for 2 kHz LFP signals while further optimizing performance by avoiding the high dynamic power of megahertz-range clocks.

Thermal safety requires limiting temperature rise. To prevent brain tissue damage, the Food and Drug Administration (FDA) restricts local temperature increases to 1 °C. For wireless implants, this sets a maximum power budget of approximately 7.7 mW [7,47]. The implant-side of NeuroGator operates at a mere 51 nW, providing substantial power and thermal dissipation headroom for other implantable BCI decoding functions within this safety constraint. As summarized in Table 1, the core feature-extraction logic consumes only 51 nW per channel and occupies a silicon area of $0.006{\mathrm{mm}}^{2}$.

## 4. Conclusions

This paper proposes and validates NeuroGator, a low-power gating system using LFP-based brain-state estimation. NeuroGator addresses power-consumption challenges in implantable brain–computer interfaces. The system replaces the high-power always-on operation typical of asynchronous BCIs. It uses a multi-level gating architecture with on-chip silence detection, transition recognition, and active state confirmation. The hardware intercepts 69.4% of invalid data. On the edge side, DualGRU collaboration further reduces the portion of data transmission, accounting for 12.6% of the total. The system cuts overall power consumption by 82%, while maintaining s brain-state estimation accuracy of above 95%. Implemented in a 180 nm CMOS process, the hardware core uses only 51 nW and occupies 0.006 mm^2^. This makes NeuroGator one of the most advanced asynchronous brain–computer switches. While the MAV-based detector offers extreme power efficiency, its reliance on amplitude thresholds makes it sensitive to certain low-frequency artifacts. For example, EMG interference during vigorous movements presents a challenge for purely threshold-based gating. In addition, we recognize that our current validation relies on a specific reach-to-grasp task. The LFP population dynamics in this dataset demonstrate robust gating performance. However, the system’s generalizability across diverse motor paradigms remains a limitation that needs further investigation. Hardware optimization also remains a key objective. Integrating the MAV logic directly into the AFE could potentially eliminate unnecessary ADC sampling power during resting states. This would provide a path toward even greater energy efficiency. Taken together, this work provides an important theoretical basis and technical paradigm for developing the next generation of implantable neural interfaces, characterized by long battery life, miniaturization, and high intelligence. Building on these advancements, future work will further explore the deep fusion design of ultra-low-power circuits and algorithms to enhance robustness against physiological noise and diverse task requirements.

## Figures and Tables

**Table 1:** Performance and architectural comparison of asynchronous BCI gating systems.

Work	Ours	[8]	[11]	[18]	[46]	[44]
Signal modality	LFP	LFP-BP	MUA	SBP	SBP	MUA
Process On Chip	MAV	Bandpower	RMS	Integration	Peak Detect	NEO + RMS
State Estimate	DualGRU	GRU	TCN	-	SSKF	GRU
Clock Freq (kHz)	80	-	400	500	80	800
Technology (nm)	180	180	180	180	180	180
Supply voltage (V)	1.8	1.8	1.8	1.55	0.625	1.8
Area per channel (mm^2^)	0.006	0.09	0.03	0.07	-	0.02
Power per channel (nW) ^†^	51	91.87	630	212	3660	4900

^†^ TCN: Temporal Convolutional Network; SSKF: steady-state Kalman filter.

## Data Availability

The benchmark dataset analyzed in this study is publicly available on G-Node at https://doi.org/10.12751/g-node.f83565 [37]. Other relevant data are available from the corresponding author upon reasonable request.
